# Deformation and Force Chain of Two-Dimensional Granular Systems under Continuous Loading

**DOI:** 10.3390/ma16155441

**Published:** 2023-08-03

**Authors:** Fanxiu Chen, Yuxin Liu, Yuan Wang, Yanji Gu, Yang Yu, Jie Sun

**Affiliations:** 1 School of Science, Qingdao University of Technology, Qingdao 266520, China; lyx1439396593@163.com (Y.L.); 15253011069@163.com (Y.W.); 13016771918@163.com (Y.G.); y842294015@163.com (Y.Y.); 2 School of Civil Engineering, Qingdao University of Technology, Qingdao 266520, China; qdlgdxsj@163.com

**Keywords:** granular system, force chain, contact force, digital image correlation (DIC), soil modeling

## Abstract

A continuous loading experiment of a two-dimensional granular system was carried out and the experimental data were obtained by digital image correlation (DIC). The deformation field of the granular system and the changing laws of the deflection angle and coordination number of the granules on force chains with time were obtained. Based on the granule element method, the quantitative calculation of contact force was realized, and the internal force chains of the granular system were identified. The effects of contact force between granules and mechanical parameters on the evolution of force chains in a two-dimensional granular system under line loads were analyzed. The formation, evolution, and reconstruction of force chains in a granular system during loading, as well as the influence of the force chain network evolution on the macroscopic mechanical properties of granules were discussed. The experimental results indicated that the evolution of force chains was directly related to the number, geometric properties, and permutation distribution of granules in direct contact with the external load.

## 1. Introduction

Granular systems are ubiquitous in daily life, industrial production, and geological processes. All matters such as powder particles, sands, and even ice cubes that reach tens of meters in the planetary ring, of which the spatial scales span from micrometers to meters, belong to granules. Many natural phenomena and practical problems are directly related to granular systems, including avalanches, sandstorms, quicksand, debris flow, high-temperature gas-cooled reactor core granule flow and granular spallation target of advanced nuclear fission energy systems. Many major engineering issues are also closely related to granular matters, including the slow settlement of railway ballast, the long-term stability of high-rise buildings and rockfill dams under cyclic loads (e.g., shearing), the safe landing of lunar rover, and the prediction and prevention of landslides. After the Wenchuan earthquake in 2008, due to the broken surfaces, the mountains in this area became prone to frequent large-scale debris flows for more than ten years or even longer. The composition and motion features of the broken and scattered materials are essentially different from the laws of previous debris flows, which will seriously undermine the economic development and the safety of people’s lives and property in Southwest China according to our existing experiences. This sparks the exploration of granular systems that have not been well understood but are related to the national economy and the people’s livelihood. Any new knowledge and findings about the mechanical properties of granular systems will produce significant economic and social benefits.

Sun [1] simulated the two-dimensional granular systems under loads in the static granular stack state with the discrete element method and carried out a quantitative analysis on the characteristics of force chains and the distribution rules of contact force. Iikawa [2] experimentally studied the statistics of force-chain evolution in a vertically tapped two-dimensional granular packing by using photoelastic disks and found that a negative correlation between the increasing of interparticle forces and the disordering of force-chain orientations. In the study of force chains in three-dimensional frictionless granular systems based on experiment, Zhou [3] measured the contact force and contact angle between micro-droplets with a high-resolution fluorescence confocal microscope, determined the existence of three-dimensional force chains, and established the relationship between force chain network and elastic response between macro-granules.

The photoelastic method, due to the intuitive and quick observation of the internal stress of photoelastic granules, has become the main method to inspect the mechanical properties of granular systems. Kollmer [4] carried out cyclic loading experiments on two-dimensional photoelastic granule layer, with the results showing that the force chain network structure is the key to analyzing the pressure on a single granule, and the total pressure on a granule is related to the intermediate centrality value of all contact forces of the granule extracted from the force chain network.

With the development of computer and image processing technology, digital image correlation (DIC) [5,6,7,8,9] has been introduced to the research of granular matter mechanics. Chauve [10] studied the evolution of local non-uniform strain field around intragranular cracks in polycrystalline ice at the beginning of the tertiary creep based on DIC for the first time. Hurley [11] improved the granular element method (GEM) and described the transfer of internal force of granules with arbitrary shape or texture and opaque granules with DIC. Subsequently, Chen [12] and Marteau [13] combined DIC with GEM in their experiments, calculated the internal contact force of two-dimensional opaque granular systems and the granule kinematics parameters, and analyzed the identified force chain network and its evolution.

Grain size has a certain effect on the mechanical properties of objects. Many scholars have studied the effects of particle size on the mechanical properties of objects [14,15,16]. Lakirouhani [17] studied the strength, physical, and engineering index parameters of dolomitic rocks with the same mineral composition but different grain size, found that there are significant positive correlations between grain size and uniaxial compressive strength, point load strength, Brazilian strength, and average Young’s modulus. Tian [18] used biaxial simulation of different particle shapes to explore the evolution of force chains and micro and micro-macro parameters at the critical state, and quantified particle shapes by length–diameter ratio.

With the advancement of science and technology, some high-tech contactless methods have also been applied to detect the mechanical properties of granular systems, such as acoustic emission [19], scanning electron microscope [20], X-ray computed tomography [21], and nuclear magnetic resonance [22]. However, these methods have not been widely implemented due to their strict requirements for environment and equipment, long scanning time, and especially the impossibility of in situ loading.

Previous research has shown some achievements in the meso-scale measurement of granule size, shape, porosity, etc. and in the identification of force chains. However, these achievements are still preliminary. There is a lack of meso-scale qualitative, quantitative, and systematic experimental research on granular systems. The experimental data on the meso-scale mechanical parameters, especially on the meso-scale porosity, granule displacement, rotation angle, coordination number, and the relationship with the macro scale are absent. However, such data are vital for the analysis of the mechanical properties and stability of granular systems. 

In order to investigate the influence of interparticle contact force and mechanical parameters on the evolution of force chain of two-dimensional particle system, a study was conducted to apply the DIC technique to a Schneebeli model [23] and to observe deformation and contact chains developed during a loading. In this paper, the effects of contact force between granules and mechanical parameters on the evolution of force chains in a two-dimensional granular system under load are explored by combining DIC with dynamic experiment of granular systems. The formation, evolution, and reconstruction of force chains in a granular system during the loading process are analyzed, and the experiment of a cylindrical granular system [24] under external loads based on DIC was designed to observe the internal movement of the granular system, identify the internal displacement of the granular system and the distribution of contact force between granules, extract the internal force chains in the granular system, analyze the evolution law of force chains, and explore the influence of the evolution of force chain network on the macro mechanical properties of granules. 

## 2. Calculation of Contact Force between Granules Based on DIC

Most of the traditional material measurement methods are based on contacts, which feature many limitations, such as limited measurement range, incomplete data, single direction, and vulnerability to disturbance. Currently, the non-contact measurement of granular systems based on the digital image correlation method (DIC) is adopted, which can achieve better quantitative analysis of the mechanical properties of granular systems owing to the advantages of the full-field, non-contact, highly automatic, and low experimental environment required by DIC method.

The granule element method [25,26,27,28] is a method for calculating the contact force between granules by using Newtonian mechanics and the momentum balance theory. This method solves the unknown forces by extracting the permutation distribution and coordinate position of each granule, establishing the torque equilibrium equation, force balance equation, and momentum balance equation composed of the known and unknown forces for each granule, and importing the force generated by the known external load on the granule into the balance equations. Combined with DIC, the displacement and strain values of each point in the granular system are obtained, and the experimental results are input into the granule element method to calculate the contact force between the granules in the granular system.

## 3. Loading Experiment of the Granular System

### 3.1. Experimental System

Considering that the inevitable rotation of spherical granules in the experiment will lead to the loss of their surface speckles and thereby inaccessibility to the further motion information, a cylinder loading experiment was designed to observe the two-dimensional circular granular system formed by the neatly arranged cylinder ends from the front. The steel cylinders had different diameters d = 3 mm, 4 mm, 5 mm d = 3 mm, and the same length h = 20 mm, and the Young’s modulus was 200 GPa. Since the material used in the experiment was made of steel, the pressure on the material was far less than its strength and was not enough to deform the granules. A concave groove was self-developed as the container of the granular system. In use, the length of the internal backing plates and the rear screws were adjusted according to the granule size, so that the granules could be placed in a single layer, which was convenient to observe the deformation of the two-dimensional circular granular system from the front. 

**Figure 1:**
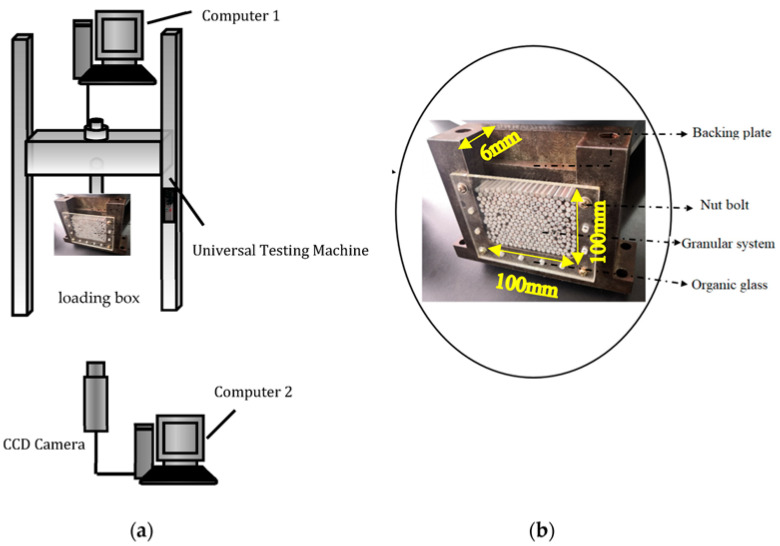
(**a**) Schematic diagram of experimental system and (**b**) loading box.

**Figure 2:**
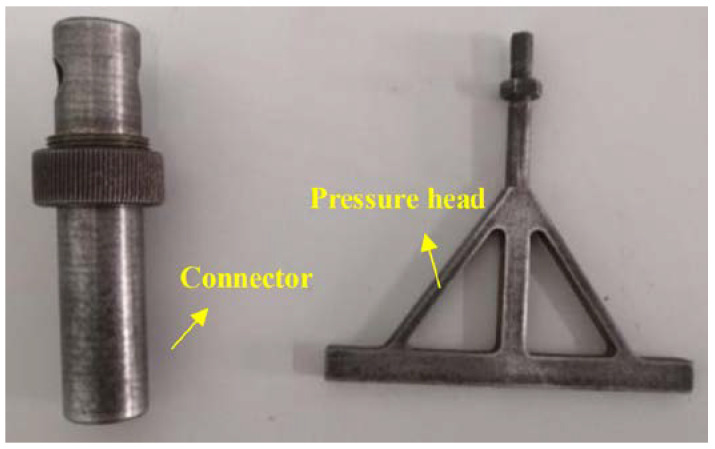
Physical drawing of connector and pressure head.

The experimental system, as shown in Figure 1, comprised a CCD camera, a universal testing machine, a loading box, specimens, and two computers. The loading box was the container box for the granular system. The CCD camera (Product from Basler in German. Model: scA1600-14 fm) has an acquisition frequency of 2 fps. During the experiment, the universal testing machine (Brand: HengYi Precision Instrument. Model: HY-0580) was connected with the special pressure head to provide line load for the granular system. The testing machine controls the pressing speed of the pressure head and records the change of the contact force at the bottom of the pressure head with the pressing displacement in real time. In order to study the deformation of the granular system under line load, the pressure head and its connector were self-developed, as shown in Figure 2. The connector is the part connecting the testing machine and the pressure head. Designed with nuts, the connector can be firmly connected to the testing machine, ensuring the smooth progress of the experiment. It is time-consuming to design and manufacture the pressure head for the experiment, since it shall be designed repeatedly in combination with factors like the testing machine, loading box, and granule size, and shall have high precision and stiffness. The designed pressure head had a small width of 6 mm and could ensure the uniform line load application on the granular system in the test. In order to explore the factors influencing the stability of the granular system, the evolution of the internal force chains in the granular system was further studied. Line load application experiments were carried out on cylinders with loading speeds of 2 mm/min. The initial state required that the granules be randomly placed, undisturbed, and loose. By analyzing the image captured by CCD, the porosity was 15.02% at the initial state.

Considering that the collected images will express light and shade variation affected by the AC power supply of the laboratory lighting equipment, which may cause certain errors in the measurement results, the strip light source was used as the supplementary lighting to avoid insufficient illumination, flicker, and other problems. The dimensions and properties of tools and materials used in the experiment are shown in Table 1.

### 3.2. Loading Experiment of Granular System

The granules were scattered on the ground and sprayed with a combination of black and white matte paints. The granules were fully sprayed with white matte paint at first, and then randomly sprayed with black matte paint to generate randomly distributed speckles on the surface. 

Since granules of different sizes were used in the experiment, the internal depth of the concave groove had to be adjusted to ensure that the granular system was placed in a single layer and to realize the external load experiment of the two-dimensional granular system. The internal depth of the concave groove could be controlled by adjusting the internal backing plates in the concave groove with cushions and screws. The concave groove with specimens was placed in the loading area, and the universal testing machine was started to lower the pressure head down to a millimeter above the granular system, waiting for loading. 

During the experiment, a gradienter was used to level the experimental platform, concave groove, camera, and pressure head, to ensure the level and stability of the experimental equipment. The aperture and focal length of the camera were adjusted according to the brightness of ambient light, and DC light source was used as the supplement lighting source to ensure the appropriate brightness and sufficient clarity of the collected images. To avoid human error, the experimental platform was kept clean and tidy, and acts such as touching the camera lens or transparent glass, which may lead to defects in the images collected, were prohibited. 

**Figure 3:**
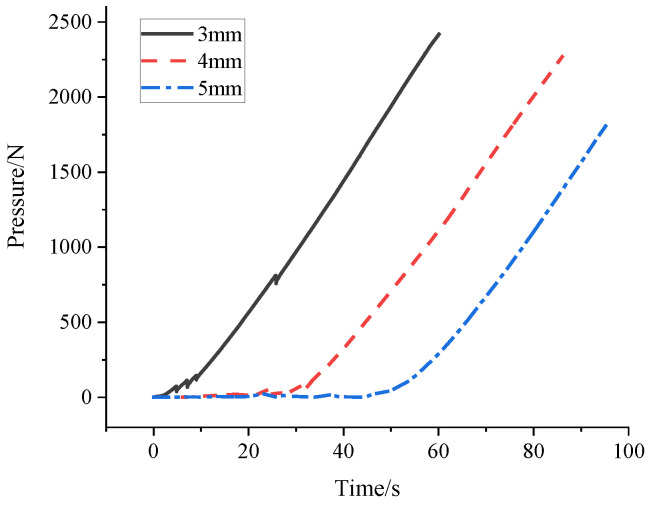
Changes in external pressure of different diameter granular systems with time.

Before the experiment, an image for the granular system without load was collected as the reference for data processing. When the universal testing machine was loading the granular system, the camera was turned on for image acquisition. A continuous rise of the force value displacement curve of the universal testing machine indicated an adequately squeezed granular system. At this time, the testing machine was stopped from pressing immediately to avoid damage. The camera acquisition was stopped as well. The images collected during the experiment and the pressure data of the testing machine were saved then. Figure 3 shows the pressure curve during the loading process of the different diameters granular system. 

### 3.3. Experiment Results and Analysis

#### 3.3.1. Deflection Angle Analysis

A granular system is composed of a large number of randomly stacked discrete granule units, and its stability is related to each granule. The external force disturbs the system, and the internal granule interaction bears the external force and maintains the balance of the system with it. This chain structure formed by the connected granule interaction is called a force chain, which plays a role of maintaining the stability of the granular system. The arrangement of granules in natural state features randomness and non-uniformity, but the internal force chains of a granular system are constantly evolving under an external load. 

During the loading of a granular system, the external force disturbs the system and the internal granule interaction bears the external force and maintains the balance of the system together with it. This chain structure formed by the connected granule interaction is called a force chain, which is divided into strong and weak types. In the study of force chain structure by Sun et al. [1], the angle criterion of forming force chain was proposed to determine a complete force chain. During the downward transmission of the force, due to the external load along the vertical direction, it will be hard to continue the force transmission when the transmission deviates from the vertical direction to a certain angle threshold, and the force chain can easily break when the transmission on the force chain exceeds the above threshold. Therefore, the transmission direction of force within a certain threshold range will be conducive to the generation and maintenance of a force chain. 

**Figure 4:**
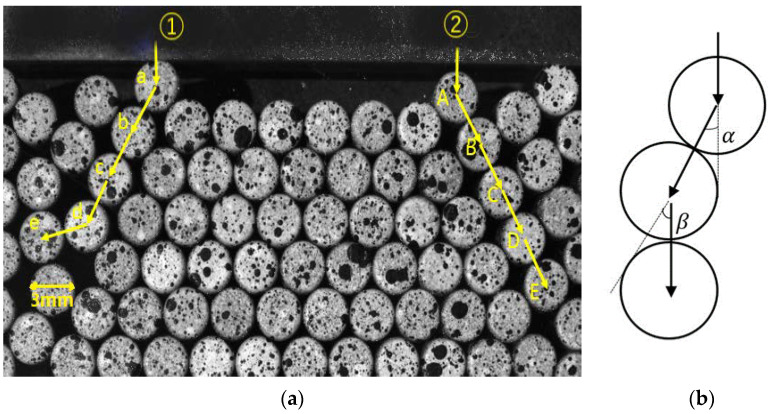
Schematic diagram of (**a**) two force chain ➀ and force chain ➁ (**b**) deflection angle.

Under the action of the vertical line load, the granular system as a whole showed a downward movement trend. Due to the closed state of the left and right sides and bottom of the concave groove, the granular system presented slow squeezing and compaction during the continuous pressing of the pressure head. In the system, two strong force chains with obvious linearity connected by the mutual squeezing of granules are identified, namely force chain ➀ and force chain ➁. The granules on the force chains were named a–e and A–E from top to bottom. As shown in Figure 4a, the force chains mainly extended along the collimation line and expanded in a tree shape. As shown in Figure 4b, the deflection angle refers to the included angle between the normal of the contact surface of adjacent granules (i.e., both 𝛼 and 𝛽 are deflection angles), which reflects the deflection amplitude of a force chain and can quantitatively describe the collimation and transmission form of a force chain. The sum of deflection angles refers to the sum of the deflection angles of each granule on a force chain. The calculation of the sum of deflection angles is helpful to explore the time of force chain break and the evolution trend of the force chain.

#### 3.3.2. Change of Deflection Angles of Granules on Force Chains

**Figure 5:**
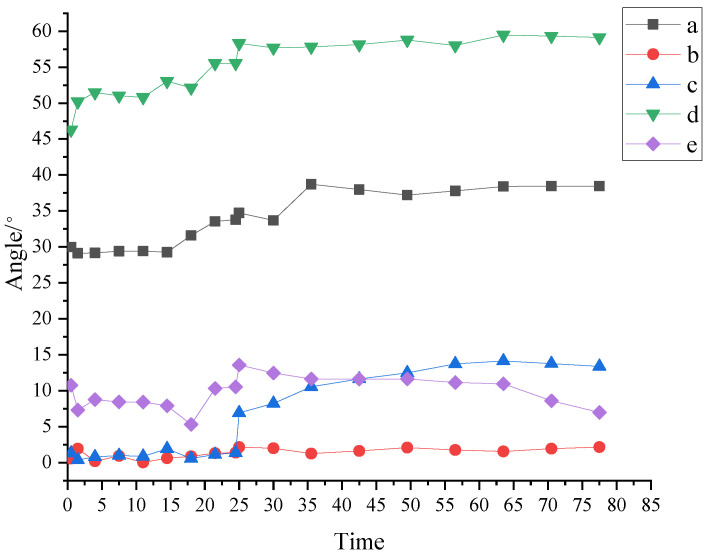
Change of deflection angles of granules on force chain ➀.

By binarizing the image and extracting the edges of particles, the center coordinates of the particles can be obtained. The deflection angle can be calculated based on the line connecting the centers of adjacent particles. Software programming can automatically achieve the calculation of the deflection angle. During force chain evolution, the granules on the two force chains showed constant sliding, rolling, and rubbing, which made the deflection angle change constantly. At the same time, the force chains were destroyed and reconstructed. Therefore, there must be a certain threshold range of the change of deflection angle, within which the force chain is less likely to break, and beyond which the force chain transmitting force will become unstable and easy to break. Figure 5 shows The deflection angles of granules on the force chain ➀ and force chain ➁ were calculated and recorded to analyze the influence of the deflection angle on the evolution of force chains.

From the evolution process of force chain ➀, the deflection angle of granule d is the largest one, followed by granule a, and the deflection angles of the other three granules are smaller than 15°. Granule b shows good collimation and no rotation of over 2°.

(1) In 0–20 s, the force chain is formed and stabilized. In 20–28 s, the deflection angle of each granule on the force chain increases. At 25 s, a substantial relative rotation occurs between granule c and granule d, resulting in a sudden increase in the deflection angle. The deflection angle of granule c on force chain ➀ surges from 1.37° to 6.94°, indicating a change of 5.57° and a great change rate of 406.4%. The deflection angle of granule d is up to 58.3°, with a change of 12°. The change between granules c and d also leads to the buckling of force chain ➀ at 25 s. The force chain is broken, with energy dissipated, and then reconstructed into a new force chain.
(2) It can also be seen from Figure 4a that the first four granules a, b, c, and d keep good collimating transmission at the beginning, and the force chain is continuously strengthened during the constant squeezing and compacting, subsequently becoming more stable and less prone to fracture. However, granule e shows a large deflection angle and poor collimation, so buckling fracture is easy to occur at the connection of granules d and e during the strengthening of the force chain. The deflection angle of granule e begins to decrease from 25 s. The force chain breaks at the connection of granule d and granule e, because of the excessive deflection angle at the connection of the two granules. The force chain is reconstructed into a secondary force chain, which continues to support the strong chain.
(3) In 28–63.5 s, the deflection angle of each granule on the force chain is basically stable with slight fluctuations, indicating that under the action of the line load, the breaking and reconstruction of the force chain are completed in a short time. The granular system is continuously compressed, resulting in the continuous reduction of internal pores, and the constant strengthening of the reconstructed force chain.

**Figure 6:**
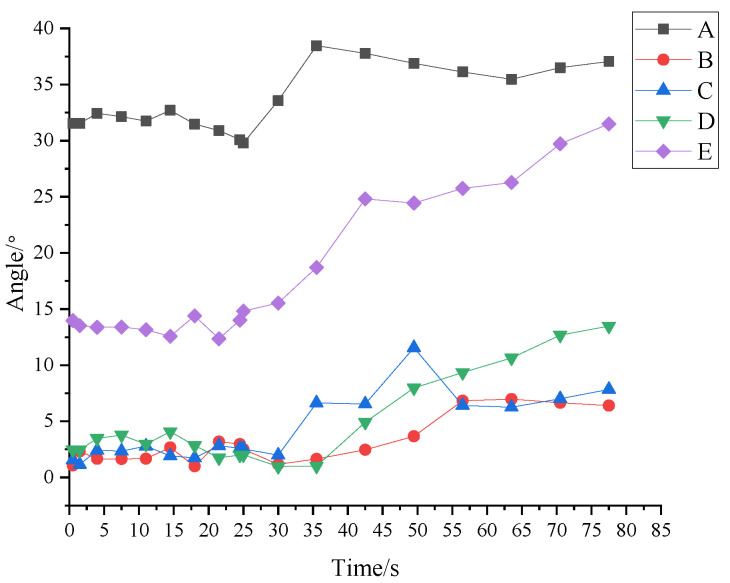
Change of deflection angles of granules on force chain ➁.

As shown in Figure 6, the deflection angle of granule A on force chain ➁ is the largest one, followed by granule E, and the deflection angles of the other three granules are less than 13°.

(1) The deflection angle of each granule on force chain ➁ fluctuates slightly before 30 s. During this period, the force chain presents good collimation and is formed and strengthened.
(2) From 30 s, the deflection angles of granules begin to change, and the deflection angles of many granules keeps increasing until the end of loading. It is worth noting that the deflection angle of granule C decreased by 6° in 50–57 s, indicating that the granule rolled rapidly in unit time. However, after 57 s, the deflection angle of granule C still tends to increase, which means that the force chain did not break at granule C.

Table 2 summarizes the deflection angles when the force chains in the granular system with different granule sizes are broken. It was found that granules with different sizes have quite different instantaneous deflection angle thresholds, and the bigger the deflection angle, the more easily the force chain will develop into a secondary force chain supporting the strong chain. For granules with different sizes, the larger the granule size, the greater the instantaneous change of deflection angle when the force chain breaks.

#### 3.3.3. Sum of Deflection Angles of a Force Chain

According to the analysis of the deflection angle of each granule on the two force chains, the granules at which the force chains break can be accurately identified, and with the deflection angles and the changes of deflection angles given, the quantitative analysis of the evolution of the force chains is realized. However, the data are not enough to analyze the time when the internal force chains of the system are broken or the angle thresholds. Therefore, the law of change for the sum of deflection angles of the force chains with time was analyzed for a more comprehensive exploration of the force chain evolution process of development–strengthening–break–reconstruction.

**Figure 7:**
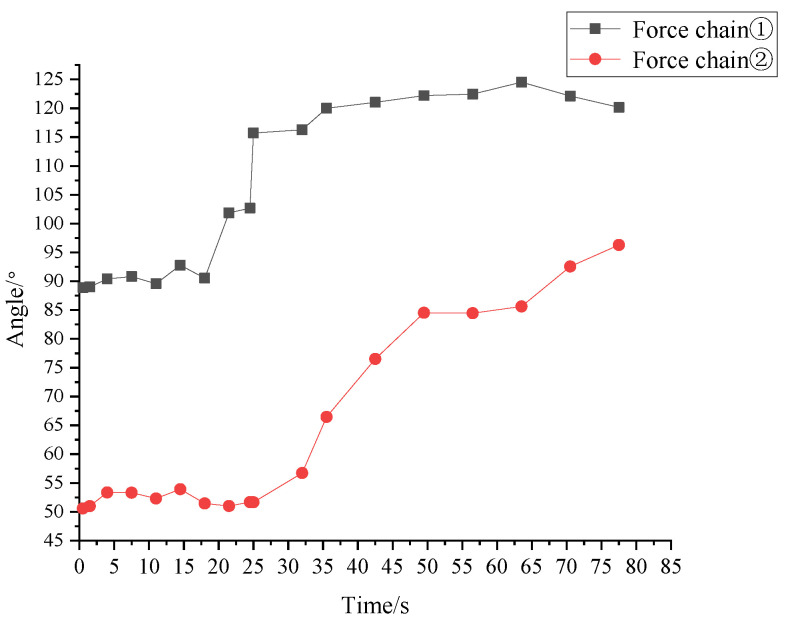
Change of the sum of deflection angles of granules on the two force chains.

As shown in Figure 7, force chain ➀ has a large transmission bifurcation at the tail, so that the sum of its deflection angles is significantly higher than that of force chain ➁. Compared with force chain ➀, the direction of force chain ➁ indicated good collimation. Both force chains present a sharply increased sum of deflection angles in 20–28 s. The break of force chain ➀ leads to the surge of the sum of deflection angles as great as 115°, indicating that the break and reconstruction of the internal force chain ➀ did not affect the evolution of the force chain ➁ during this period. 

Comparing the overall changes of the sum of deflection angles of the two force chains, force chain ➀ is in the continuous development and strengthening stage in the first 20 s, when the force on the granules on the force chain increases gradually, and the force chain becomes a strong one with the enhancement of the interaction between granules. At 20 s, due to the transmission bifurcation at the tail, the sum of deflection angles of the force chain changes obviously, and the integral and individual granules rotates greatly, resulting in the break of the force chain; from 20 s to 28 s, the sum of deflection angles of force chain ➀ rises abruptly, the change of the sum of deflection angles reached 115° due to the break of force chain ➀, and the internal force chain ➀ is broken and reconstructed; after 28 s, the sum of deflection angles becomes stable, which is mainly because that force chain ➀ is reconstructed under the action of the vertical load, and granules on it are compacted by each other; only at 60 s did the deflection angle of the force chain fluctuate slightly due to the evolution of other force chains. 

As shown in Figure 7, before 30 s, the sum of deflection angles of force chain ➁ was kept at about 50°, and the internal force chain ➁ was in a stable formation stage without too much angle fluctuation; at 30 s, the inflection point of the sum of deflection angles of force chain ➁ appeared, but the force chain showed good collimation throughout the loading process, and did not break as the sum of deflection angles kept increasing linearly, indicating that the relatively uniform deflection of granules on force chain ➁ after 30 s did not cause the buckling failure of the force chain, which was still a strong chain.

#### 3.3.4. Coordination Number

**Figure 8:**
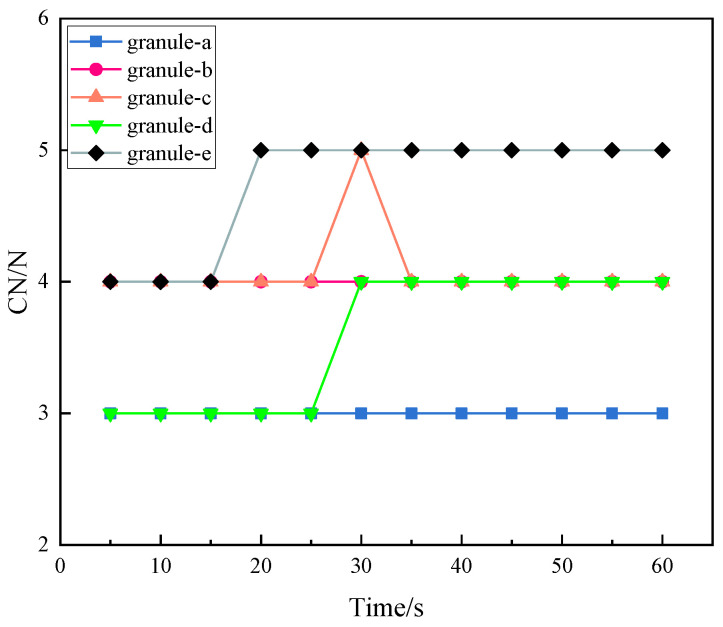
Change of coordination numbers of granules on force chain ➀.

**Figure 9:**
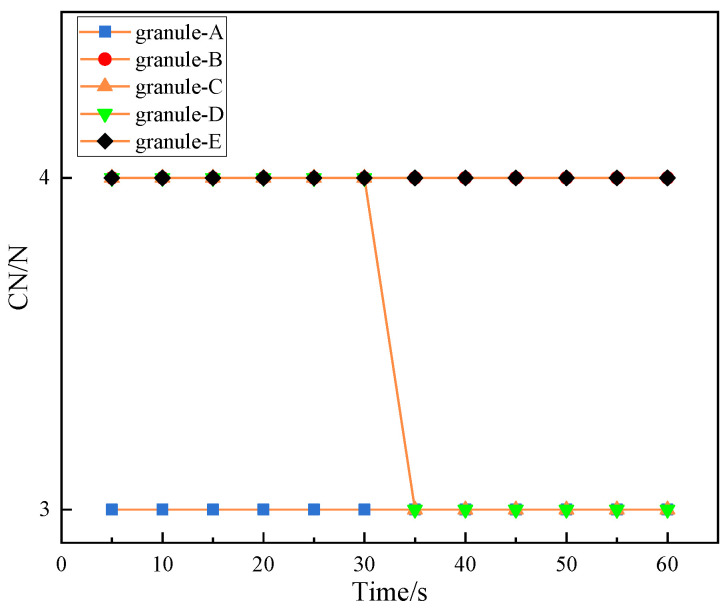
Change of coordination numbers of granules on force chain ➁.

Coordination number (CN) refers to the number of granules in contact with the current granule. It is one of the major geometric characteristics of granular systems, and is closely related to the material, size, and gradation of granules. The summary of the coordination numbers of granules on a force chain is helpful to analyze the evolution law of the force chain. Figure 8 and Figure 9 show the changes of the coordination numbers of granules on force chain ➀ and force chain ➁.

Figure 8 shows the change of the coordination numbers of granules on force chain ➀ with time. The coordination numbers of granules do not change from 0 s to 15 s. When t = 20 s, granule e begins to move, and its coordination number rises from 4 to 5, which is kept until the end of the experiment. In 25–35 s, the force chain changes greatly. In this period, the coordination number of granule c increases from 3 to 4 and then back to 3. The increase of the coordination number indicates that the granule around particle c becomes dense, which supports and strengthens the force chain ➀. It indicates that the force chain is strengthened during 30–35 s, and then the coordination number is reduced to 3 due to buckling failure at particle c. Combined with the analysis of the deflection angle of force chain ➀, it can be seen that the force chain changed at 25 s, resulting in the fluctuation of the coordination numbers of granules on it. In the later stage, buckling occurred at granule c due to the continuous strengthening of the force chain.

Figure 9 presented the change of the coordination numbers of granules on force chain ➁. As shown, the coordination numbers of two granules changes. When t = 35 s, the coordination numbers of granules C and D on force chain ➁ decreases from 4 to 3. Combining the deflection angle, force chain ➁ begins to deflect due to interference in 30–35 s under the load. When t = 35 s, granule C and granule D are moved away from their original contact granules due to interference and the force chain is strengthened in this process.

#### 3.3.5. Displacement Field Analysis

The two signs of force chain fracture in granular systems are deflection angle and displacement. Excessive deflection angle or displacement can both lead to force chain fracture. In order to explore the force chain evolution of the granular system under line load, the sequence images of the granular system collected by CCD camera during the loading process are analyzed with digital image correlation software, with the displacement diagram obtained. A two-dimensional granular system formed by the cylinders with a diameter of 3 mm was selected for the loading experiment, in which the loading speed was 2 mm/min and the image acquisition frequency was 2 fps. 

**Figure 10:**
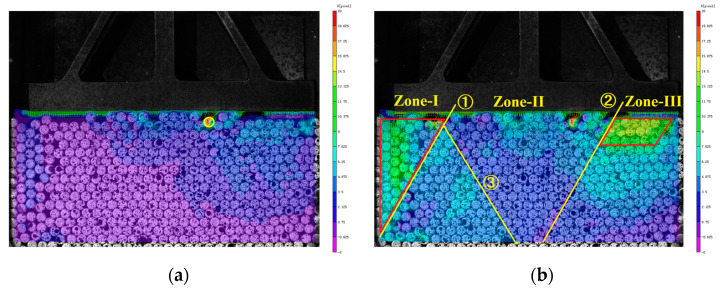

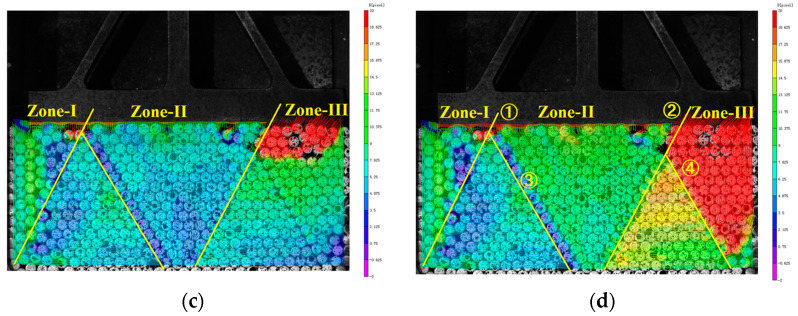
Displacement distribution. (**a**) Vector diagram at 10 s; (**b**) Vector diagram at 15 s; (**c**) Vector diagram at 20 s; and (**d**) Vector diagram at 25 s.

As shown in Figure 10a, at 10 s, the top granules show displacement first due to the vertically downward squeezing from the pressure head. In the vector diagram, there is an obvious granule (at the yellow circle mark in Figure 10a); the granule has no direct contact with other granules vertically above it, but directly interacted with the granules on its left and right horizontally. It can be inferred that the granule rotated under the squeezing of the left and right granules. Since there is no granule above it, the local porosity is larger, and the granule rotates clockwise because of the mutual dislocation of the granules on its left and right. The clockwise rotation makes it move upward in the left half and downward in the right half, which is also proven by its vector length. The interaction between the granule and the left granule is greater than that between it and the right one, which explains the reason for the simultaneous upward and downward displacement of the same granule. 

Overall, the local granules on the upper right move upward by about 4 pixels, while the displacements of granules in other areas were less than 2 pixels. The displacement of granules on the upper right is significantly greater than the overall average displacement of granules in the granular system, which is closely related to the number and position of granules in direct contact with the pressure head. Considering the practical reality in engineering, the slope, road, foundation, and others do not all have synchronous and horizontal contact surfaces when subject to external loads. Therefore, before the design experiment, the original granules are not placed very horizontally and densely. Under the action of the external force, granules on the upper right of the granular system are not in contact with the pressure head, and there are more granules on the right of the upper granules in contact with the pressure head than those on the left, resulting in greater displacement on the right part.

As shown in Figure 10b, the granular system is further squeezed and compacted under the action of the external line load at 15 s, and the granules in direct contact with the pressure head in the middle continued to move downward, showing a tree-like development starting from the compressed granules. The upper half of the left and right parts have large pores, and the middle granules were relatively dense, so the movement forms of granules in different parts of the granular system varied markedly. 

In Figure 10b, the granular system is divided into the left Zone I, the middle Zone II, and the right Zone III by line ➀ and line ➁. Zone II is further divided into the left and right parts by line ➂. Zone I is an inverted triangle and Zone III is a block. Line ➀ is an obvious granule gap strip from the right top to the left bottom, with a large porosity. The gap strip is located at the contact between the loose and dense granules, forming a shear zone. The granules in Zone I on the left of the shear zone are sparse with a large downward displacement, and those in Zone II on the right of the shear zone are dense with a small downward displacement. Granules in the lower part of the right Zone III of the granular system are dense, all of which have a coordination number of six. Granules in the upper part of the right Zone III are sparse and moved downward after the start of loading process. Their compactness and stability increase as they move lower, and their displacement shrinks gradually. Granules in Zone II show small displacement as a whole, but there is still an obvious shear zone (located at the line ➂) inclined to the lower right. The granules on the left of the line are sparse, with bigger displacements, and those on the right are dense, with smaller displacements. 

As shown in Figure 10c, when t = 20 s, the middle dense part in Zone II gradually extends to the left, and the left Zone I shrinks, with displacement around 8–13 pixels. Under the action of the load, the frame area in the upper half of Zone III begins to show large displacement, reaching above 40 pixels. The upper right area suddenly presents a large and multi-directional displacement during the loading due to large pores. The rolling and dislocation between granules in this area results in the loss of local displacement information, marking the beginning of the break of the force chain network in this area.

As shown in Figure 10d, under the continuous pressure, the granular system in the middle Zone II is gradually squeezed at 25 s, and the shear zone ➂ becomes increasingly obvious. The rolling of granules in the shear zone ➂ makes the internal energy of the granular system released. The granules in the shear zone ➂ are squeezed towards the less constrained regions on both sides, so that the displacements in these regions further increased. Zone II is divided into two inverted triangular parts by the shear zone ➃ caused by granule dislocation. The dislocation between granules reduces the coordination number of granules in the shear zone ➃ from 6 to 5, resulting in larger pores between granules. Granules in the middle Zone II are dense, with a downward displacement of more than ten pixels. The lower granules move to the looser areas on the left and right under continuous pressure to alleviate and release the internal energy. Zone I is in a narrow triangular shape and moves upward. Due to the contact between the upper granules and the pressure head, the granules show deflected displacement and spiral rolling in the upper left.

## 4. Extraction and Analysis of Force Chain in Granular System

### 4.1. Granule Contact Force Analysis

**Figure 11:**
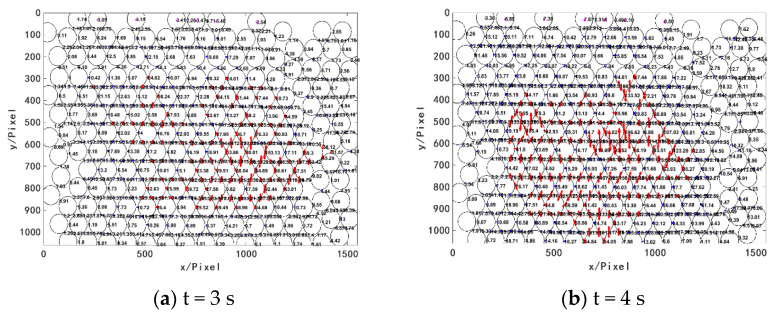

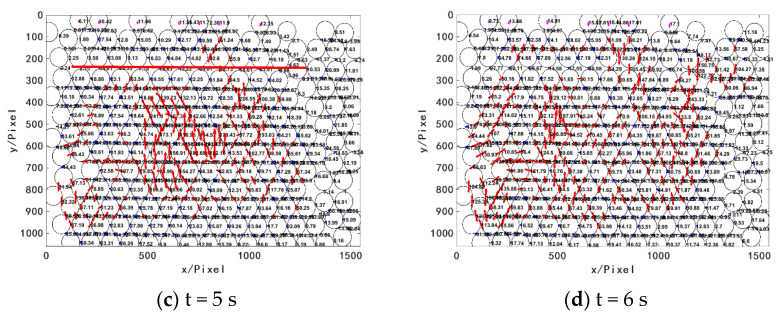
Distribution of contact force between granules (Unit: N).

MATLAB was used for identification of images collected by the experiment. The arrangement structure of the whole granular system was calculated based on the center of granules and the number of contact points. The granule momentum balance and Newton’s law of mechanics were programmed to calculate the contact force between the granules in the granular system combined with the displacement and strain data obtained by the digital image correlation method, as shown in Figure 11. The size of contact force was marked on the contact point. The length of the line segment was used to represent the size of the contact force and the inclined direction of the line segment was used to represent the direction of the contact force. The blue line segment means contact force was small, and the strong force chain is marked with a red line segment. The main purpose of these graphs is to demonstrate the evolution law of the force chain network and the changes in the strong chain.

When t = 3 s, the pressure head begins to have contact with the granular system, and the force on the top granules is 1.74–3.54 N. At this time, the granular system, under small external load, is unstable, and the force chains are being formed. The middle area of the granular system is less affected by boundary contact. The contact force between granules is close to the loading direction (vertical direction), dominating the formation of the initial force chains in the granular system. 

When t = 4 s, with the continuous downward pressure from the pressure head, the force on the top granules is larger, and the interaction force between granules in the granular system increases. The granules are distributed in a tree structure extending downward from each granule in the top layer. As the number of top granules in contact with the pressure head increases, the contact force is constantly recombined during force transmission, and the force chains are in the stage of continuous formation and gradual strengthening. 

When t = 5 s, due to factors such as arrangement, bite, and loading of the granular system, interlaminar dislocation occurs on a straight line parallel to the *x*-axis when y = 240 pixel, resulting in the break of the vertical force chains there, as shown in Figure 11c. A small lateral force supports the force chain, but an excessive lateral force leads to the break of the force chain and the dislocation of the granular system. 

When t = 6 s, with the downward load increased, the granular system is further squeezed and compacted, the internal granules are rearranged, the interaction between granules is further strengthened, and the contact force becomes larger. A new force chain network is formed in the system and the reconstruction of the force chain is realized.

The analysis of contact force between granules in the granular system under line load is summarized as follows:

(1) The evolution of force chains has a direct relationship with the number, geometric properties, and permutation distribution of granules in direct contact with the external load and the force chains develop in a tree structure starting from the granules in direct contact with the external load.
(2) The direction of the force chains is basically consistent with the action direction of the external load, and the tangential force will lead to the break of the force chains.
(3) The porosity of the granular system has a direct impact on the structure and stability of the force chains, and the granules in the granular system usually move to the less constrained regions with larger porosity.
(4) For a granular system with good bite and initially complete, the granule dislocation starts from the position with a larger granule gap in the upper part. Under the continuous external load, the dislocation between granules continues to extend throughout the whole granular system, forming a shear zone in the system, so that the internal energy of the system is released, which is conducive to the stability of the force chain network.
(5) The local regional fracture of the granular system often occurs in the existing fractures. Under the action of the external load, the dislocation of the granular system will further increase until the local system shows overall obvious displacement or sudden separation.

### 4.2. Evolution of Force Chain Network

The granular system with a granule size of 3 mm and a large porosity is used to explore the evolution and development of its force chain network. Under the action of line load, the top granules in the middle contacted with the pressure head first since there are certain pores in the left and right parts of the granular system, and the force chain presents a tree structure and extended downward from the contact points. Under the action of line load, the randomly distributed and uneven granules show the contact of multiple granules as seen at the beginning, presenting a tree-like force chain. With the progress of the experiment, the number of granules in contact with the pressure head increase. The number and arrangement of granules in contact with the pressure head affects the formation of the force chain structure and constantly disturbs the formed force chain structure. Due to the change of the granular system structure, the force chains keep evolving. In this process, the force chain network presents different network forms.

**Figure 12:**
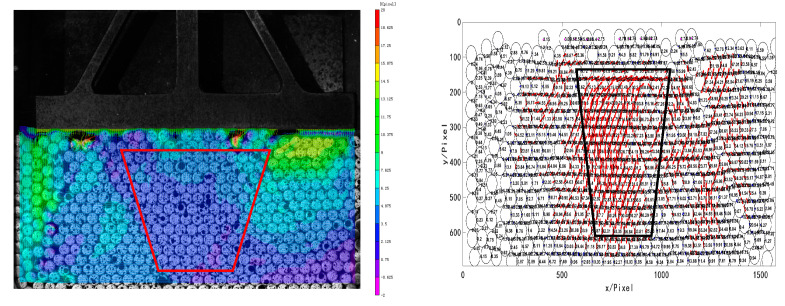
Displacement cloud chart and diagram of contact force between granules at 10 s.

Figure 12 shows the displacement of the granular system and the contact force between the granules at 10 s. It can be seen from the displacement cloud chart that the granules in contact with the pressure head moved first. Under the downward pressure, the tree-like force chains starting from the granules on the top layer disturb each other in the process of downward transmission, making the force chain structure in the granular system change constantly. According to the diagram of the contact force between granules, the contact force in the middle develops vertically and downward under the interference of granules on both sides. The force chain network consists of tree-like network structures with the top granules as the start. Under the mutual interference of many force chains in the granular system, a complex network structure was formed, and the strong force chain in the middle is trapezoid, as shown in the black wireframe area in the figure.

**Figure 13:**
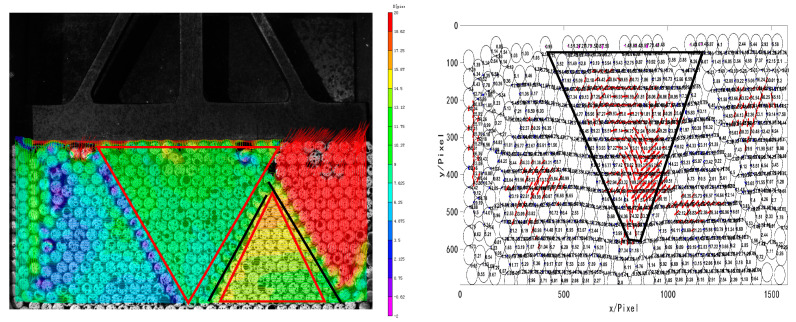
Displacement cloud chart and diagram of contact force between granules at 20 s.

As shown in Figure 13, during the later development of the force chain network, when t = 20 s, the top granules under pressure are all in the horizontal position and in contact with the pressure head. The granules in the local areas on the left and right are not closely arranged due to the bite between the granules, with certain pores. The continuously squeezed middle granules expanded to both sides, causing the local granules with certain pores on both sides to displace. In this way, the energy accumulated inside is released. As observed in the experiment, under the action of the line load, an obvious shear zone is formed between dense and loose granules. When the dislocation expands further and the upper part is not constrained enough, the local fracture of the force chain network occurs at the granules in the loose area, and the dense granules in the middle gradually developed into a larger triangular force chain network structure, as shown in the black wireframe area in the figure.

## 5. Conclusions

Granular systems are ubiquitous in daily life, industrial production, and geological processes, and the study of granular systems can play a preventive role against natural disasters such as mudslides and earthquakes. In this paper, we loaded the granular system by line loading, and investigated the breakage and reorganization evolution law of the force chain. Then, a study was conducted to apply DIC technique to a Schneebeli model and to observe deformation, contact chains developed during a loading.

(1) In this research, the loading test of a two-dimensional granular system under line load was carried out. The deformation information of the granular system was analyzed by DIC, and the contact force between granules was calculated quantitatively.
(2) With the changes of deflection angle, coordination number, and other parameters with time, each parameter on the formation and evolution of force chains was influenced, and the effect of the evolution of force chain network on the macroscopic mechanical properties of granules were hence explored.
(3) The evolution of force chains was directly related to the number, geometric properties, and permutation distribution of granules in direct contact with the external load. The force chains developed in a tree structure with a strong anisotropy, and later inclined to the force direction. The granules in the granular system moved to the less constrained regions with large porosity, and the evolution of force chains included stages of formation, strengthening, fracture, reconstruction, and stabilization.

## Figures and Tables

**Table 1:** Tools and materials used in the experiment.

Name	Quantity	Dimensions	Attribute
Cylinder	Sufficient	d = 3 mm, 4 mm, 5 mm; h = 20 mm	Steel
Pressure head	One	Bottom: 95 mm × 6 mm	Steel
Connector	One	d = 20 mm, h = 114 mm	Steel
Backing plate	Several	Different thickness	Steel
Matching bolt and nut	Several	M3, M4, M5, M6	Steel
Organic glass	Several	Thickness: 0.5 mm, 1 mm	Plastic
Matte paint	Two bottles	N/A	Black, white (liquid)

**Table 2:** Statistics of angles with force chains broken.

Test Number	Diameter (mm)	Deflection Angle (°)	Instantaneous Angle Change (°)
1	3	12.2	3.8
2	3	8.4	4.4
3	4	7.8	5.4
4	4	9.4	5.5
5	5	6.5	5.57
6	5	5.8	6.45

## Data Availability

The datasets generated during or analyzed during the current study are available from the corresponding author on reasonable request.
